# Transient Loss‐Induced Non‐Hermitian Degeneracies for Ultrafast Terahertz Metadevices

**DOI:** 10.1002/advs.202304972

**Published:** 2023-10-28

**Authors:** Weibao He, Yuze Hu, Ziheng Ren, Siyang Hu, Zhongyi Yu, Shun Wan, Xiang'ai Cheng, Tian Jiang

**Affiliations:** ^1^ College of Advanced Interdisciplinary Studies National University of Defense Technology Changsha 410073 P. R. China; ^2^ Institute for Quantum Science and Technology College of Science National University of Defense Technology Changsha 410073 P. R. China

**Keywords:** active non‐Hermitian metasurface, loss‐induced EP modulation, ultrafast terahertz metadevice

## Abstract

Non‐Hermitian degeneracies, also known as exceptional points (EPs), have attracted considerable attention due to their unique physical properties. In particular, metasurfaces related to EPs can open the way to unprecedented devices with functionalities such as unidirectional transmission and ultra‐sensitive sensing. Herein, an active non‐Hermitian metasurface with a loss‐induced parity‐time symmetry phase transition for ultrafast terahertz metadevices is demonstrated. Specifically, the eigenvalues of the non‐Hermitian transmission matrix undergo a phase transition under optical excitation and are degenerate at EPs in parameter space, which is accompanied by the collapse of chiral transmission. Ultrafast EP modulation on a picosecond time scale can be realized through variations in the transient loss at a non‐Hermitian metasurface pumped by pulsed excitation. Furthermore, by exploiting the physical characteristics of chiral transmission EPs, a switchable quarter‐wave plate based on the photoactive metasurface is designed and experimentally verified and realized the corresponding function of polarization manipulation. This work opens promising possibilities for designing functional terahertz metadevices and fuses EP physics with active metasurfaces.

## Graphical abstract

An active non‐Hermitian metasurface with a loss‐induced parity‐time symmetry phase transition for ultrafast terahertz modulation is demonstrated. The eigenvalues of the non‐Hermitian transmission matrix undergo a phase transition under optical excitation and are degenerate at EPs in parameter space. By exploiting the physical characteristics of chiral transmission at EP, a switchable polarization manipulation metadevice is designed.

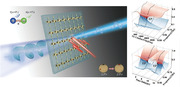

## 1 Introduction

The successful commercialization of 5G has sparked a research boom in the next generation of wireless communication. Terahertz waves, as a candidate frequency band, can transmit high‐speed information,^[^ ^1^, ^2^, ^3^ ^]^ and comes with countless applications in biochemical sensing,^[^ ^4^, ^5^ ^]^ security screening,^[^ ^6^ ^]^ and holography.^[^ ^7^ ^]^ However, the scarcity of corresponding functional devices limits the commercial development of terahertz technology. Fortunately, the metasurface emerges as a novel planar device with periodic arrangements of subwavelength meta‐atoms that can be exploited for various terahertz functionalities, including frequency filtering,^[^ ^8^ ^]^ focusing,^[^ ^9^, ^10^ ^]^ phase control,^[^ ^11^, ^12^ ^]^ beam steering,^[^ ^13^, ^14^ ^]^ and polarization conversion.^[^ ^15^ ^]^ Since the proposal of the pioneering work combining semiconductors with metal metasurfaces,^[^ ^16^ ^]^ dynamic terahertz modulation using optical,^[^ ^17^, ^18^ ^]^ electrical,,^[^ ^19^ ^]^ thermal,^[^ ^20^ ^]^ magnetic,^[^ ^21^ ^]^ and mechanical^[^ ^22^, ^23^ ^]^ methods have been vigorously developed. The dynamically tunable nature overcomes the drawbacks of passive metasurfaces in immutable size, greatly enriching the versatility of terahertz devices based on metasurfaces. Especially for all‐optical control, modulation with the contactless and ultrafast response can be achieved. Nevertheless, terahertz functional metadevices based on transient perturbation mostly focus on amplitude and phase modulation.^[^ ^24^, ^25^, ^26^, ^27^ ^]^ The inefficiency of the ultrafast metadevice hinders it from exploring more other utilitarian applications. Perhaps one need to find new ways to improve the efficiency and versatility of metadevices. A better solution to this challenge is to find novel physical mechanisms.

Research into non‐Hermitian physics in photonics has recently made rapid progress owing to extraordinary phenomena such as unidirectional invisibility,^[^ ^28^ ^]^ asymmetry switching,^[^ ^29^, ^30^ ^]^ enhanced sensing,^[^ ^31^ ^]^ and phase topology in encircling exceptional points (EPs).^[^ ^32^, ^33^ ^]^ Unlike a closed system described as a Hermitian Hamiltonian in which energy conservation is strictly obeyed, a non‐Hermitian system is open with complex eigenvalues.^[^ ^34^ ^]^ In general, building non‐Hermitian systems requires balancing the relationship between the gain and loss of materials,^[^ ^35^ ^]^ which is challenging to find the gain materials for metal metasurfaces. One can create virtual gain by altering the loss of resonant modes to study the physical properties of broken parity‐time (PT) symmetry and phase transition at EPs in a metasurface system.^[^ ^36^ ^]^ An important concern is EPs in non‐Hermitian systems, which are at the critical point of PT symmetry and broken PT symmetry, where the eigenvalues are identical and the eigenvectors become completely degenerate.^[^ ^37^ ^]^ The non‐Hermitian degeneracies cause plentifully novel physical phenomena with enormous potential for designing high‐performance applications in functional terahertz metadevices.

A simple way to build a non‐Hermitian metasurface for terahertz modulation is to design two coupled Lorentzian dipoles composed of split ring resonators (SRRs) orientated along perpendicular directions.^[^ ^38^ ^]^ The phase transition from PT symmetry to broken PT symmetry can be induced by controlling the loss of metals and the coupling distance between two resonators. Similarly, one can construct a non‐Hermitian system by using coupled resonators with the same metal material but different dissipation losses.^[^ ^39^, ^40^ ^]^ The EP located at the metasurface can be found by adjusting the coupling distance. However, active PT symmetry breaking with tunable parameters for a terahertz metasurface is highly desirable for practical applications. Although several works have studied theoretically and experimentally the active control of the PT symmetry phase transitions,^[^ ^41^, ^42^, ^43^, ^44^ ^]^ they focused solely on the dynamical modulation of non‐Hermitian physics instead of functional terahertz metadevices based on the loss‐assisted metasurfaces.

To create non‐Hermitian metasurfaces that can be used in novel terahertz applications, we propose a non‐Hermitian system composed of amorphous germanium (Ge) film and a patterned metal metasurface and demonstrate the ultrafast dynamical modulation of PT symmetry phase transition via optical excitation. In addition, the conversion from linear to circular polarization is realized by taking advantage of the asymmetry chiral transmission near the EP. Specifically, we first design the Ge‐hybrid non‐Hermitian metasurface guided by coupling‐mode theory and present the loss‐induced PT phase transition. Terahertz time‐domain spectroscopy affords a broadband frequency range, which when combined with continuously tunable dissipated loss caused by Ge conductivity forms a two‐dimensional parameter space for identifying EPs. Moreover, time‐resolved spectroscopy provides precise time delay, enabling both ultrafast terahertz modulation and continuous variations in excited‐Ge conductivity. The eigen transmissions in experiment and simulation are degenerate and lack a chiral dimension at the EP, leading to a dramatically asymmetric characteristic that can be used for a metadevice with linear to circular polarization conversion. This work not only realizes ultrafast modulation of the non‐Hermitian phase transition but also converts chiral polarization by using properties near the EP, providing a new paradigm for designing non‐Hermitian functional terahertz metadevices.

## 2 Results

### 2.1 Engineering Active Non‐Hermitian Metasurface

**Figure 1:**
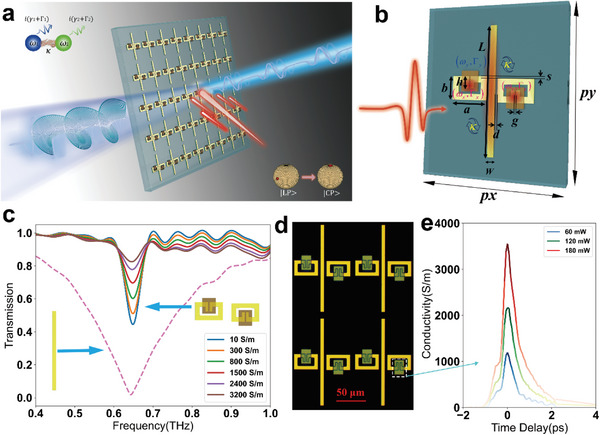
Design and manufacturing of Ge‐hybrid non‐Hermitian metasurface for optically pumped terahertz modulation. a) Artwork of applications on Ge‐hybrid non‐Hermitian metasurface with optical excitation. b) The geometric parameters of unit cell are px  =  102, py  =  157, w  =  7, a  =  41, b  =  27, g  =  2, d  =  3, h  =  7, s  =  1, with unit of µm. c) The transmission spectra of two orthogonal resonators, wherein, the dissipation loss along x direction can be tuned via Ge conductivity. d) Microscopic image of the Ge‐hybrid non‐Hermitian metasurface. e) Transient conductivities of Ge islands with various pump powers.

According to the temporal coupled‐model theory, the metasurface system composed of resonance modes in the *x* and *y* directions to incident electromagnetic wave can be described by a 2 × 2 non‐Hermitian Hamiltonian,^[^ ^41^ ^]^

(1) $$\begin{array}{c} H_{0}=\left(\begin{array}{cc} \gamma_{y}\left(\omega_{x}-\omega \right)+j\left(\gamma_{y}\Gamma_{x}-\gamma_{x}\Gamma_{y}\right)/2 & \kappa \sqrt{\gamma_{x}\gamma_{y}}\\ \kappa \sqrt{\gamma_{x}\gamma_{y}} & \gamma_{x}\left(\omega_{y}-\omega \right)+j\left(\gamma_{x}\Gamma_{y}-\gamma_{y}\Gamma_{x}\right)/2 \end{array}\right) \end{array}$$

where ω_*x*,*y*_, γ_*x*,*y*_, Γ_*x*,*y*_ are the resonance frequency, radiation loss, and dissipative loss, respectively, and κ is the coupling strength between two resonant modes. For ω_*x*_ = ω_*y*_ , one can find the EP by using $|\gamma_{y}\Gamma_{x}-\gamma_{x}\Gamma_{y}|=\;2\sqrt{\gamma_{x}\gamma_{y}}\left|\kappa \right|$, which suggests that the degenerate eigenvalues of the Hamiltonian can be built by designing the damping loss and coupling the strength of metasurface unit. For an actively controlled phase transition, semiconductor materials through optical and electrical excitation can introduce tunable dissipative loss,^[^ ^45^, ^46^ ^]^ implying a flexible terahertz modulation based on non‐Hermitian metasurfaces. Herein, we design a Ge‐hybrid non‐Hermitian metasurface whose unit cell consists of a cut wire along the *y* direction and a pair of SRRs embedded in the Ge film at gaps along the *x* direction, as shown in **Figure** **1**b and which supports two orthogonally oriented resonance modes. The resonance frequency and the loss rate of CW and SRRs can be tailored by the geometry and constituted material. The transmission spectra of two orthogonal resonators are shown in Figure 1c, where the dissipation loss of the resonators along the *x* direction can be tuned by adjusting the conductivity (Figure 1e). We conclude that the EP in the non‐Hermitian metasurface will appear upon optical excitation in the parameter space of frequency and dissipation loss caused by Ge conductivity. The conceptual application of this metadevice (Figure 1a) offers the functionality of all‐optical terahertz transmission modulation and conversion from linear to circular polarization. We fabricated this structure (see Methods), and the microscopic image of the Ge‐hybrid non‐Hermitian metasurface is shown in Figure 1d. Next, we will utilize the degree of freedom of loss, an indispensable element in non‐Hermitian physics,^[^ ^36^, ^47^ ^]^ to construct a loss‐assisted terahertz modulator.

### 2.2 Loss‐Induced PT Symmetry Breaking with Exceptional Degeneracies

**Figure 2:**
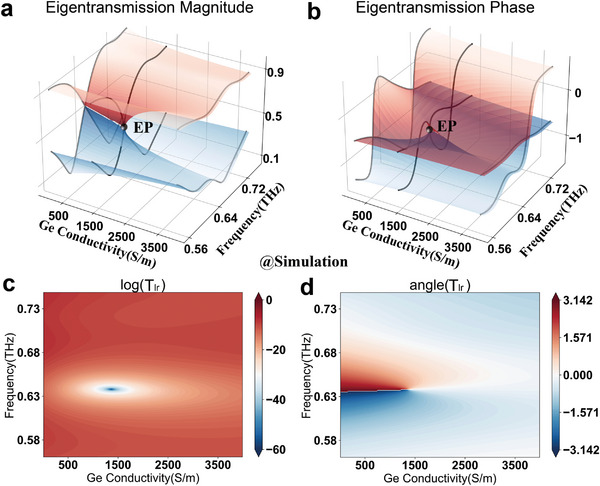
Simulation results of the Ge‐hybrid non‐Hermitian metasurface in the parameter space covered by frequency and Ge conductivity. a) Simulated eigentransmission magnitude and b) phase in the parameter space covered by frequency and Ge conductivity. Riemannian surfaces colored by red, and blue describe respectively the first and second eigenvalues of the second‐order transmission matrix. EP caused by loss occurs in the position of eigenvalue degeneracy. c) Extracted eigentransmission magnitudes (red solid lines) and phases (blue dashed lines) versus simulated Ge conductivities. The chain lines mark the frequency of 0.65 THz. The EP is found to be located at the frequency of 0.64THz and Ge conductivity of 1370 S m^−1^. d) Simulated chiral cross polarization transmission amplitude represented by unit dB (d) and e) phase represented by unit rad via sweeping Ge conductivities. Near‐zero transmission and singular phase appear at the EP position.

The non‐Hermitian Hamiltonian matrix describing the metasurface system can develop from PT symmetry to broken PT symmetry upon the relationship between modes loss and coupling. This inspired us to produce EPs by changing the material loss (i.e., the conductivity of photoactive islands). Herein we consider a 2 × 2 transmission matrix to describe the eigentransmission of the metasurface. The transmission matrix can be described as

(2) $$T\;\left(R\right)=\left(\begin{array}{cc} t_{xx}\left(R\right) & t_{xy}\left(R\right)\\ t_{yx}\left(R\right) & t_{yy}\left(R\right) \end{array}\right)\;$$

where *R* is the parameter space, and *t_ij_* is a complex transmission for polarization state *i* outgoing with state *j* incidence, {*i*, *j*} ∈ {*x*,  *y*}. The corresponding eigenvalue can be derived as

(3) $$\lambda_{1,2}=\frac{t_{xx}+t_{yy}\pm \sqrt{{\left(t_{xx}-t_{yy}\right)}^{2}+4t_{xy}t_{yx}}}{2}\;$$

where λ_1,2_ are the complex eigenvalues of the 2 × 2 transmission matrix. To find the EP in the Ge‐hybrid metasurface, we thus first perform simulations to hold the four complex transmission spectra by using finite integral technology, following which the eigenvalues of the transmission matrix as a function of Ge conductivities can be obtained. The Riemannian surfaces depict the variation of eigentransmission magnitudes (**Figure** **2**a) and phases (Figure 2b) in the parameter space covered by frequency and the conductivity. Loss‐induced EP occurs at the position of eigenvalue degeneracy in the parameter space of (*f*_*EP*, *sim*_,  σ_*EP*_) = (0.64 THz,  1370 S m^−1^) , where the Riemannian surface intersects both eigentransmission magnitudes and phases. For more details, see the extracted eigentransmission magnitudes and phases versus simulated conductivity of photoactive islands shown in Figure S1 (Supporting Information). The numerical simulation captures the entire process of loss‐induced EP phase transition. For σ < σ_*EP*_, the eigentransmission magnitudes cross and the system Hamiltonian can be described as PT symmetry. When the case is σ > σ_*EP*_, implying higher loss than coupling, the two eigentransmission magnitudes do not intersect, and the Hamiltonian can be described as breaking PT symmetry. Especially for critical coupling (i.e., σ  = σ_*EP*_ ), the eigentransmission magnitudes and phases intersect at the same position where the EP arises, which suggests that the loss‐induced EP phase transition can be used to design EP‐based active metadevices. In addition to the degeneracy of transmission eigenvalue, another characteristic of EP is the polarization eigenstate coalescence, which leads to singular behavior in the chiral transmission spectra through the non‐Hermitian metasurface. Going a step further, we numerically perform a chiral transmission spectrum *t_lr_* as a function of Ge conductivities, as shown in Figure 2c,d. The four chiral transmission spectra can be obtained from linear polarization transmission,

(4) $$\begin{array}{c} \left(\begin{array}{cc} t_{rr} & t_{rl}\\ t_{lr} & t_{ll} \end{array}\right)=\frac{1}{2}\;\left(\begin{array}{cc} t_{xx}+t_{yy}+i\left(t_{xy}-t_{yx}\right) & t_{xx}-t_{yy}-i\left(t_{xy}+t_{yx}\right)\\ t_{xx}-t_{yy}+i\left(t_{xy}+t_{yx}\right) & t_{xx}+t_{yy}-i\left(t_{xy}-t_{yx}\right) \end{array}\right) \end{array}$$

where *t_lr_* is defined as the complex transmission for left‐circular polarization (LCP) outgoing with right‐circular polarization (RCP) incident, and so on for other chiral components. To better highlight the EP, we define the transmission magnitude as log (*t_lr_*) =  10log(|*t_lr_* |) in dB units. The near‐zero transmission *t_lr_* means the polarization eigenstate is RCP. In order words, there is no LCP component when RCP is incident, so only RCP transmission occurs. In addition, a full 2π phase accumulates upon encircling the EP. The singular behavior of magnitude and phase is unique to the EP in the non‐Hermitian metasurface system, which is a more intuitive way to observe EPs.

**Figure 3:**
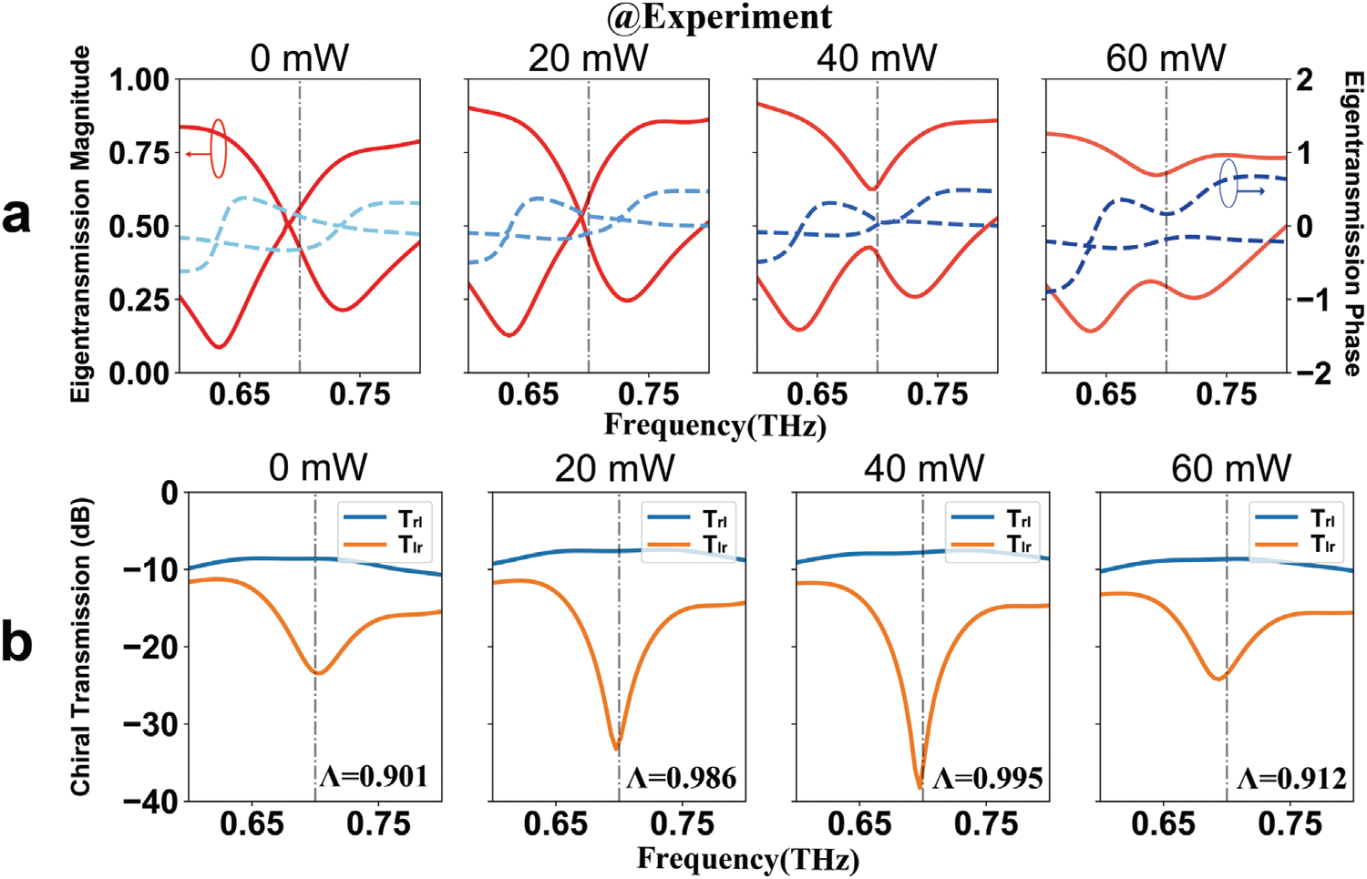
Experiment measurement of EP in non‐Hermitian metasurface with various pump powers. a) The eigentransmission magnitudes (red solid lines) and phases (blue dashed lines) at the optical pump powers of 0, 20, 40, and 60 mW. b) Corresponding chiral cross‐transmission magnitude *T_rl_* and *T_lr_*, whereas *T_lr_* is suppressed with respect to *T_rl_*. The chain lines mark the frequency of 0.7 THz. The characteristic of asymmetric transmission implies that the eigenstate near EP. It can conclude that the loss‐assisted EP occurs between the pumps of 20 and 40 mW and is closer to the pump of 40 mW from the results of eigenvalues and chiral transmissions.

Subsequently, experiments involving the eigenvalues of the Ge‐hybrid metadevice system with various optical pump powers have been carried out to observe EPs in the non‐Hermitian metasurface. A homemade optical pump terahertz‐probe (OPTP) system was used to obtain terahertz transmission spectra through the metasurface as a function of pump power. Four linearly polarized transmission components can be obtained by rotating the sample and polarizer (see Figure S4, Supporting Information). Then the eigentransmission values and chiral polarization transmission spectra are calculated by using Equations (3) and (4). According to the simulation results, the EP appears at the eigenvalue degeneracy ( λ_1_ = λ_2_ ) accompanied by zero chiral transmission ( |*t_lr_* | =  0). We experimentally tried various eigentransmission magnitudes and phases (**Figure** **3**a) with pump powers of 0, 20, 40, and 60 mW. The eigentransmission magnitudes intersect at the pump power of 20 mW but separate at the pump power of 40 mW, along with the eigentransmission phases from separation to intersection. By reason of its sensitivity, the EP in the experiment is difficult to check strictly, but we conclude that the EP appears at the pump power between 20 and 40 mW in our experiment. Specifically, the EP occurs precisely at the maximally asymmetric transmission.^[^ ^48^ ^]^ The asymmetric chiral transmission through the metasurface can be characterized by an “asymmetry factor”,

(5) $$\Lambda \;=\frac{{\left|t_{rl}\right|}^{2}-{\left|t_{lr}\right|}^{2}}{{\left|t_{rl}\right|}^{2}+{\left|t_{lr}\right|}^{2}}\;$$

When Λ = ± 1 corresponds to the EP condition, which is the maximally asymmetric transmission. The chiral transmission magnitude of *t_lr_* decreases first and then recovers along with the increasing pump power, as shown in Figure 3b. The maximally asymmetric transmission and near‐zero transmission magnitude of *t_lr_* simultaneously indicates that EP is close to the pump power of 40 mW.

### 2.3 Ultrafast Evolution Dynamics of Non‐Hermitian Phase Transition

**Figure 4:**
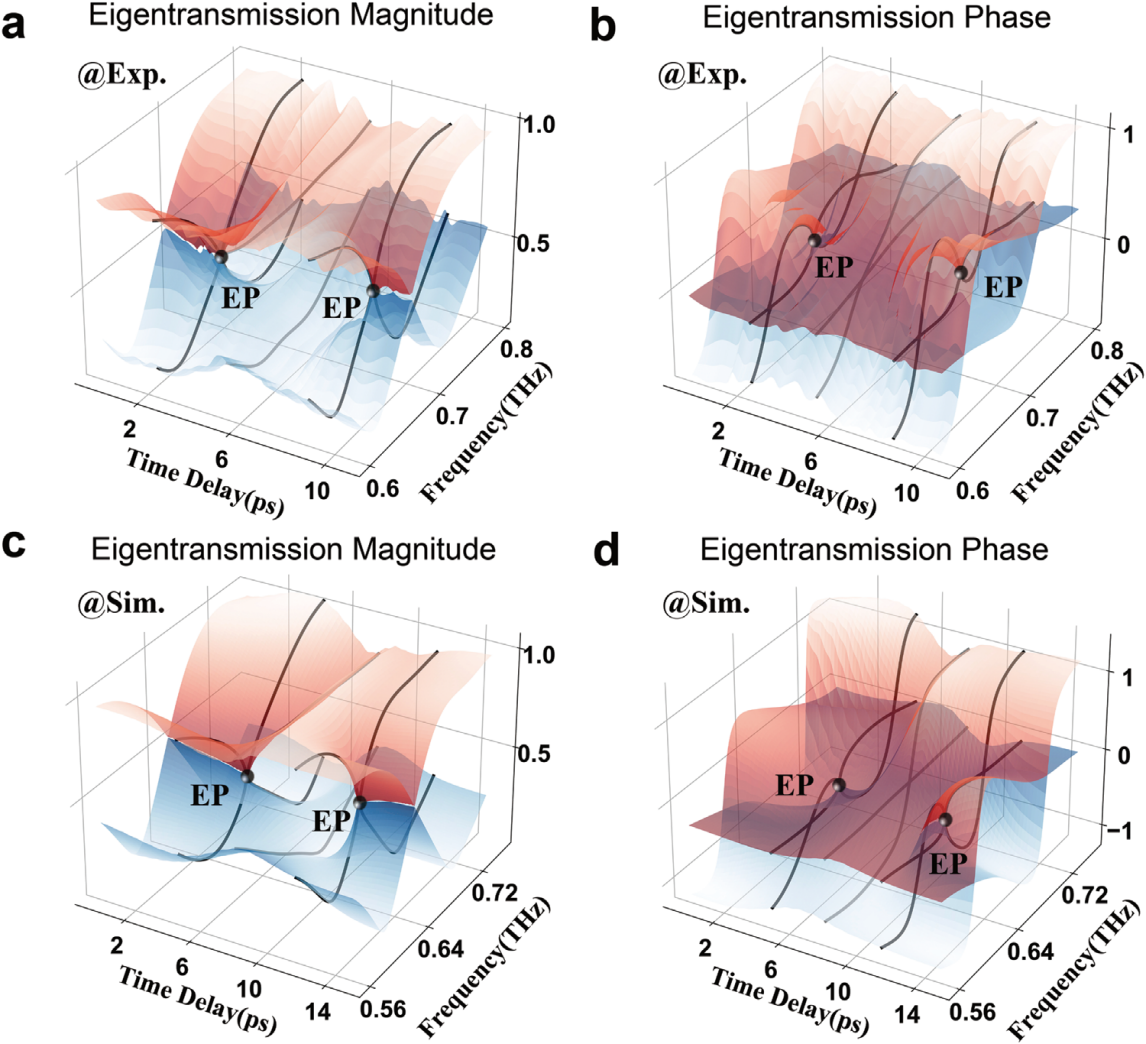
Temporal evolution process of the eigentransmission magnitude and phase in the non‐Hermitian Ge‐hybrid metasurface system implemented by optical pump terahertz probe technology. a) Experimentally measured eigentransmission magnitude and b) phase in the parameter space consisting of the frequency and the time delay. c) Numerical simulation of eigen transmission magnitude and d) phase in the parameter space consisting of the frequency and the time delay. The first and second eigenvalues of the second‐order transmission matrix are described by the red and blue Riemannian surfaces, respectively. EPs appear at the intersection of the eigentransmission magnitudes and phases, where the eigenvalues degenerate.

With the exploitation of OPTP technology, not only ultrafast dynamic terahertz modulation but also continuous conductivity changes can be achieved by varying the pump‐probe delay. Thus, the temporal evolution of the eigentransmission magnitude and phase in the Ge‐hybrid non‐Hermitian metasurface can be implemented for studying EPs. It is worth noting that the terahertz time‐domain pulse lasts 1–2 picoseconds but extends when passing through a resonance cavity of the metasurface, showing an exponential oscillation in the time domain.^[^ ^49^ ^]^ . Another important piece of information is that the relaxation time of amorphous Ge under femtosecond laser pumping is also a few picoseconds.^[^ ^50^, ^51^ ^]^ Therefore, the maximum modulation effect can be obtained only when the pump pulse strictly matches the time‐domain signal of the terahertz resonance frequency. Due to the smaller bandgap (0.66 eV) of Ge vis‐à‐vis the excitation energy (1.55 eV), femtosecond photoexcitation of amorphous Ge results in electronic transitions followed by collision loss and recombination that return electrons to the ground state in a short time, leading to a transient conductivity. Ultrafast modulation derives from the transient loss of Ge islands whose duration is comparable to that of the terahertz pulse, thus we no longer care whether the loss value is quasi‐steady‐state relative to terahertz wave, as Ge islands composite metal cells form a time‐varying non‐Hermitian metasurface that dynamically tailors terahertz waves with excitation pump. By moving the relative position of the pump delay stage to the terahertz pulse, the ultrafast dynamic behavior can be measured. **Figure** **4**a,b show respectively the experimentally measured eigentransmission magnitudes and phases in the parameter space consisting of frequency and time delay. Two EPs appear in the ascending and descending stage of Ge carrier concentration, respectively, throughout the entire ultrafast modulation process. The eigentransmission magnitudes and phases at EPs can be seen by cutting Riemann surfaces. A time difference of 6.3 ps is found in the experiment between the appearance of two EPs. Moreover, we take the transient conductivity of amorphous Ge film extracted from the experiment into the time‐domain simulation to numerically calculate ultrafast terahertz EP modulation. The transient transmission of Ge film that varies with time delay is measured by using OPTP at the pump power of 280 mW (1.4 mJ cm^−1^). The transient Ge conductivity can then be calculated by,^[^ ^52^ ^]^

(6) $$\Delta \sigma \;=\frac{\varepsilon_{0}c\left(1+n_{s}\right)}{d}\;\frac{\Delta T}{T}$$

where ε_0_ is the vacuum permittivity, *c* is the speed of light, *n_s_* is the refractive index of the substrate, and *d* is the thickness of the Ge film. Δ*T*/*T* is the differential transmission amplitude of pure Ge film, and Δσ  =  σ − σ_0_ is the differential conductivity. Here, the initial conductivity σ_0_ is set as 10 S m^−1^. The numerical eigentransmission magnitudes and phases as a function of time delay are described respectively in Figure 4c,d. The simulation results of EP phase transition evolution are consistent with the experimental results. A slight difference in time delay at two EPs between the simulation (6.6 ps) and experiment (6.3 ps) comes from the thickness error of the Ge film, which influences the extracted Ge conductivity. In addition, the experimental results are affected, notably the adhesion state of sputtering Ge film, the refractive index of the substrate, and the processing accuracy of the metal microstructures. More details of the dynamical results appear in Figure S2 (Supporting Information). Although slight differences occur, the numerical evolution is consistent with the experimental results. Substantially, ultrafast terahertz modulation on the picosecond time scale is achieved by pumping a Ge‐hybrid non‐Hermitian metasurface, which ensures phase evolution for EP switching between PT symmetry and broken PT symmetry.

### 2.4 Asymmetric Chiral Transmission with Nontrivial Topological Phenomena

**Figure 5:**
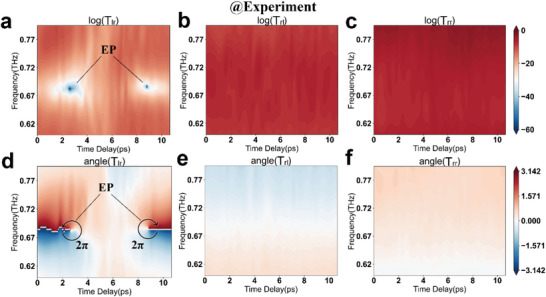
Temporal evolution dynamics of the chiral transmission in the Ge‐hybrid metasurface implemented by optical pump terahertz probe technology. a,b,c) The experimentally measured transmission magnitude of *T_lr_* (a), *T_rl_* (b), and *T_rr_* (c) as a function of pump‐probe time delay. d,e,f) The experimentally measured transmission phase of *T_lr_* (a), *T_rl_* (b), and *T_rr_* (c) as a function of pump‐probe time delay. A full 2𝜋 phase encircling accumulation and zero transmission magnitude of *T_lr_* confirm the position of EP. The metasurface system at EP shows asymmetric transmission, log(*T_lr_*) ≠ log(*T_rl_*), and only the RCP component is transmitted for the input state with RCP, abs(*T_lr_*)  =  0,  abs(*T_rr_*) ≠ 0.

The eigenmodes of non‐Hermitian metasurface merge into a chiral polarization mode (LCP or RCP) as the eigenvalues degenerate at the parameter space. In the studied Ge‐hybrid non‐Hermitian metasurface, the eigenstates degenerate into RCP at the EP, which appears as a zero LCP transmission component upon RCP input. Accordingly, we experimentally extract the chiral transmission magnitudes and phases over time. Two singularities of transmission magnitude (i.e., EPs described in **Figure** **5**a) are seen in the plot of log(*t_lr_*) with various time delays. The phenomenon suggests a missing dimension in the reduced eigenpolarization at chiral EPs when a terahertz pulse passes through the non‐Hermitian metasurface. In addition, the counterclockwise and clockwise phase accumulations of 2𝜋 occur at EPs, showing a robust nontrivial phase topology. The opposite phase circulation originates from the two processes of carrier excitation and relaxation in amorphous Ge islands (photoactive conductivity increases and then decreases, referring to Figure 1e, with the same loss condition at EPs. Notably, the frequency domain space cannot be used as a tunable parameter to design the adiabatic process by encircling EPs.^[^ ^53^, ^54^ ^]^ Nevertheless, EPs at non‐Hermitian metasurface still exhibit numerous unique physical properties at the macro level. It is obvious that there is a significant asymmetric transmission near the EP position upon comparing *t_lr_* (Figure 5a) and *t_rl_* (Figure 5b), and only the RCP component transmits through the Ge‐hybrid non‐Hermitian metasurface when RCP is incident at EP positions upon comparing *t_lr_* (Figure 5a) and *t_rr_* (Figure 5c). The transmission magnitude and phase of *t_lr_* undergoes an ultrafast modulation of ≈10 ps, passing through two EPs before and after, and then going back to the initial state. The ultrafast modulation response is attributed to the ultrafast carrier relaxation of amorphous Ge film at the picosecond level and the absence of a significant slow light effect at the metasurface. The transmission spectra of *t_rl_* and *t_rr_* do not undergo significant modulation throughout the entire OPTP process (and so on for *t_ll_* by reason of *t_ll_* = *t_rr_*  in theory). This is interesting because the change in Ge conductivity excited by the optical pump only influences one chiral component, echoing the design of modulators with asymmetric transmission. Corresponding simulation results are shown in Figure S3 (Supporting Information). Although differences between the experiment and simulation come from the calculated Ge conductivity and substrate refractive index as previously analyzed, it does not affect the research of evolutionary dynamics in the phase transition process at non‐Hermitian metasurfaces. Thus, we have demonstrated an ultrafast dynamic process of chiral transmission in experiment and simulation, which exhibits an asymmetric transmission property accompanied by the evolution of the EP phase transition.

### 2.5 Transmission Polarization Manipulation Based on Asymmetric Chiral Features Near EP

**Figure 6:**
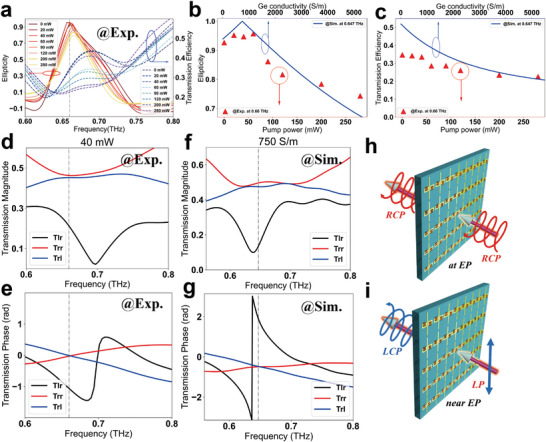
Active controlled device of polarization conversion from LP to LCP designed by asymmetric transmission characteristic near EP. a) The transmission ellipticity and efficiency as a function of optical pump powers in the case of y‐polarization incidence. The chain lines mark the frequency of 0.66 THz, where the metasurface can hold near‐perfect circular polarization while maintaining high transmission. b) Measured ellipticity (red triangle) with various pump power and simulated ellipticity (blue line) with various Ge conductivity. c) Measured transmission efficiency (red triangle) with various pump power and simulated transmission efficiency (blue line) with various Ge conductivity. d,e) Experimentally measured transmission magnitude (d) and phase (f) of *T_lr_*, *T_rr_*, *T_rl_*. The chain lines mark the frequency of 0.66 THz. f) Numerically simulated transmission magnitude and g) phase of ***T***_***lr***_, ***T***_***rr***_, ***T***_***rl***_. The chain lines mark the frequency of 0.647 THz. Two chiral transmission efficiency of the designed metadevice is equal at the operating frequency, *T_rr_* = *T_rl_* . h) Schematic diagram of RCP output for RCP input at EP. i) Schematic diagram of LCP output for LP input using chiral asymmetry near EP.

The emergence of an EP gives rise to asymmetric chiral transmission and the degeneracy of circular polarization. In addition, we use EPs to implement the functionality without chiral cross polarization, as shown in **Figure** **6**h. A conceptual study on the correspondence between incident and output light at the EP reported that completely circular polarized light is emitted when the components of the incoming LCP and RCP are in a certain ratio.^[^ ^43^ ^]^ However, setting the system's state at the EP is difficult due to its sensitivity.^[^ ^55^ ^]^ While designing a device near the EP is relatively easy. Herein, the proposed non‐Hermitian metasurface causes linear‐to‐circular polarization conversion based on the asymmetric chiral transmission near the EP, as shown in Figure 6i. As we know, linearly polarized light can be composed of two circularly polarized components. When using circular polarization basis vectors, the linear deviation along the *x* direction is expressed as (1,  1)^*T*^ (we omit the normalization coefficient of $1/\sqrt{2}$ for convenience), and the linear polarization along the *y* direction is (1,   − 1)^*T*^. With the terahertz wave incident along the *y* direction, the output status is represented as,

(7) $$T_{out}=\left(\begin{array}{c} t_{rr}-t_{rl}\\ t_{lr}-t_{ll} \end{array}\right)\;=\left(\begin{array}{cc} t_{rr} & t_{rl}\\ t_{lr} & t_{ll} \end{array}\right)\;\left(\begin{array}{c} 1\\ -1 \end{array}\right)$$

where *t_rr_* − *t_rl_* is the right‐hand component of the output and *t_lr_* − *t_ll_* is the left‐hand component of the output. Near the EP, *t_lr_* −  *t_ll_* = *t_lr_*  − *t_rr_* ≠ 0 is valid. Then only *t_rr_* = *t_rl_*  is required to achieve perfect circular polarization emission. In the case of *x* polarization, *t_rr_* =   − *t_rl_* is required. In other words, when the magnitude and phase of *t_rr_* and *t_rl_* are equal, incidence in the *y* direction produces LCP light. Moreover, conditions of the same magnitude but opposite phases of *t_rr_* and *t_rl_* need to be satisfied to produce LCP light from incidence in the *x* direction. Significantly, the proposed metadevice supports LCP light emission for incident *y* polarization, and the ellipticity and transmission efficiency can be tuned via the optical pump. We define the ellipticity of the transmission wave through the Stokes vector as,

(8) $$\xi \;=\;tan\left(\frac{1}{2}arcsin\left(\frac{2E_{x}E_{y}}{E_{x}^{2}+E_{y}^{2}}sin\delta \right)\right)$$

where δ  = φ_*y*_  − φ_*x*_. The transmission efficiency is $\sqrt{({|t_{rr}-t_{rl}|}^{2}+{|t_{lr}-t_{ll}|}^{2})/2}$. The ellipticity is larger than 0.9 at the considered frequency of 0.66 THz below the pump power of 40 mW, implying a nearly perfect LCP in the experiment, and the transmission efficiencies remain ≈0.35, as shown in Figure 6a. Optical pumping thus provides effective modulation. The extracted ellipticity and transmission efficiency (red triangles) as a function of pump power are described in Figure 6b,c, respectively. The simulation results described by blue lines demonstrate the variation well. The transmission efficiency gradually decreases with increasing pump power and Ge conductivity, due to the increase of loss, while an optimal position exists where engineering the relation between chiral transmission components will increase the maximum ellipticity. Figure 6d,e describe respectively the transmission magnitude and phase at the pump power of 40 mW, which satisfies the condition of *t_rr_* = *t_rl_*  for the outgoing LCP given *y* incidence. Although some discrepancies with the experimental results appear, the simulation spectra in Figure 6f,g confirm that the asymmetric transmission near EP can be used to design the metadevice for linear‐to‐circular polarization conversion. Of course, the optimal position should be set to the working frequency at the EP due to the maximum of |*t_lr_* − *t_ll_* | ( *t_lr_* =  0). Relatively speaking, the maximum polarization conversion efficiency can be achieved, which is the next goal of the metadevice based on EPs.

## 3 Conclusion

In this work, a Ge‐hybrid non‐Hermitian metasurface was designed and fabricated for ultrafast terahertz modulation. The proposed metadevice offers non‐Hermitian degeneracy as well as polarization conversion. We first demonstrated the loss‐assisted evolution of PT symmetry phase transition by altering the conductivity of photoactive islands in the metasurface. In particular, the eigenvalues of the second‐order non‐Hermitian transmission matrix were degenerate and accompanied by vanishing chiral transmission at the EPs. Subsequently, benefits from the relaxation properties of photoactive Ge islands, both the ultrafast PT phase transition and EPs movement measured through OPTP technology were achieved. With the exploitation of asymmetric chiral transmission near EP, we demonstrated a function of linear‐to‐circular polarization conversion as a salient example for polarization tuning, which can be generalized to other application scenarios based on non‐Hermitian metasurfaces. In addition, we numerically simulated the modulation dynamics of non‐Hermitian metasurfaces, and the simulation results matched well with the experimental dates. Unlike other studies on non‐Hermitian metasurfaces, we not only uncovered the transient evolution of EPs, but also displayed an ultrafast polarization switching by transient perturbation. This work thus provides a platform for actively non‐Hermitian chiral degeneracies and designing novel terahertz functional metadevices. We believe that the transient loss‐induced non‐Hermitian metasurface proposes a feasible method of interdisciplinary research on nonlinear optics and topological photonics and may facilitate practical applications of PT‐symmetric photonics devices.

## 4 Experimental Section

### Numerical Simulations

Numerical simulations were performed by exploitation of the Finite Integral Technology method in CST Microwave Studio software. In the simulation, the gold material was set as a lossy metal with a conductivity of 4.56 × 10^7^ S m^−1^, the quart substrate was set as a normal dielectric with a permittivity of 4. The Ge material with a permittivity of 16 possessed a time‐varying conductivity that was imported from the experimental test results, while the normalized excitation signal is also from the measured terahertz time‐domain pulse. Periodic boundaries were applied to *x* and *y* directions, and open boundary conditions were employed to z directions. The corresponding time‐domain electric field probes were used to detect the complex amplitudes of transmission (*E_x_*, *E_y_*).

### Metadevice Fabrication

A conventional microfabrication technique was used to fabricate the Ge‐hybrid non‐Hermitian metasurface on the commercially available z‐cut quart substrate with a thickness of 1 mm. A gold layer with a thickness of 200 nm was first deposited by e‐beam evaporation following the 10 nm thick chromium adhesion layer. Then the patterned structure was generated by using the standard photolithography technique with a preparational lithographic mask, followed by a lift‐off procedure. Next, a 200‐nm thick amorphous Ge layer was evaporated onto the substrate by magnetron sputtering following the second photolithography and lift‐off process. Finally, the periodically arranged Ge‐hybrid metal metasurface structure with the size of 5 mm × 5 mm was realized.

### Experimental Measurement

A homemade optical‐pump terahertz‐probe system was employed to characterize the terahertz transmission of the Ge‐hybrid non‐Hermitian metadevice. A Ti: sapphire regenerative amplifier system was employed to generate a femtosecond pulse of 1 kHz repetition at a central wavelength of 800 nm for stimulating the samples as well as terahertz generation and detection stemming from nonlinear effect of 1‐mm‐thick ZnTe 〈110〉 crystal. Then four linearly polarized transmission signals were measured by rotating the sample and linear polarizer (see Figure S4, Supporting Information). The complex terahertz transmissions as a function of frequency were obtained by the Fourier transform of the time‐domain signal and then normalized the substrate data, expressed as *T* (ω) = *E_S_* (ω)/*E_R_* (ω) . The measurement of ultrafast terahertz modulation was achieved by moving the relative position between the terahertz signal and the optical pump pulse.

## Conflict of Interest

The authors declare no conflict of interest.

## Author Contributions

W.H. and Y.H. conceived the idea. W.H., Z.R., and Y.H. designed the experiment and performed active measurements and all the simulations. S.H., S.W., and Y.H. fabricated the samples. W.H., Z.Y., and S.H. discussed and analyzed the measured data. X.C., Y.H., and T.J. supervised the theory and the measurements. W.H. prepared the manuscript with inputs from T.J. and Y.H.

## Supporting information

Supporting Information

## Data Availability

The data that support the findings of this study are available from the corresponding author upon reasonable request.
