# Supplementary material for: Transient Loss‐Induced Non‐Hermitian Degeneracies for Ultrafast Terahertz Metadevices
Source: Adv Sci (Weinh). 2023 Oct 28;10(36):2304972. doi: 10.1002/advs.202304972 (PMC10754078; doi:10.1002/advs.202304972)
Supplement: Supplementary file 1 — Supporting Information [file ADVS-10-2304972-s001.pdf]

ADVANCED SCIENCE
Open Access

Supporting Information

for *Adv. Sci.*, DOI 10.1002/advs.202304972

Transient Loss-Induced Non-Hermitian Degeneracies for Ultrafast Terahertz Metadevices

Weibao He, Yuze Hu, Ziheng Ren, Siyang Hu, Zhongyi Yu, Shun Wan, Xiang'ai Cheng and Tian Jiang**

Supporting Information

Transient loss-induced non-Hermitian degeneracies for ultrafast terahertz metadevices

Weibao He[1], Yuze Hu[,2] , Ziheng Ren[1], Siyang Hu[1], Zhongyi Yu[1], Shun Wan[1], Xiang'ai Cheng[1], Tian Jiang[*,2]*

[1] College of Advanced Interdisciplinary Studies, National University of Defense Technology, Changsha 410073, P. R. China
[2]Institute for Quantum Science and Technology, College of Science, National University of Defense Technology, Changsha 410073, P. R. China
Correspondence: Professor Yuze Hu, E-mail: hyz_yj@sina.com_
Correspondence: Professor Tian Jiang, E-mail: tjiang@nudt.edu.cn_

Numerically extracted eigentransmission magnitudes and eigentransmission phases as a function of Ge conductivities from Riemannian surfaces

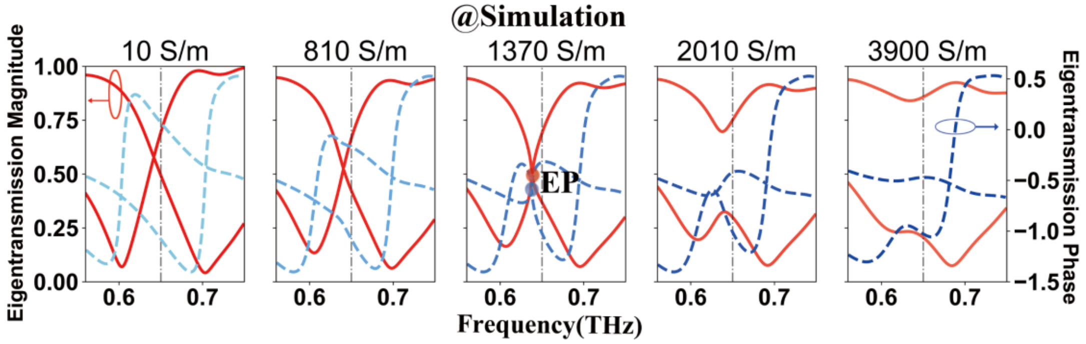

Fig. S1 Numerically extracted eigentransmission magnitudes (red solid lines) and phases (blue dashed lines) versus simulated Ge conductivities. The chain lines mark the frequency of 0.65 THz. The EP is found to be located at the frequency of 0.64THz and Ge conductivity of 1370 S/m. A crossing behavior in the magnitude and phase of eigenvalues reveals EP is located at the parameter space of $(f_{EP,sim}, \sigma_{EP}) = (0.64\,THz, 1370\,S/m)$.

Extracted eigentransmission magnitudes and phases versus time delay from experimental and simulated eigenvalue Riemann surfaces

To shed light on the temporal evolution dynamics of phase transition in non-Hermitian metasurface, we experimentally extract the eigentransmission magnitudes and phases with the time delay from 0 to 10.6 ps, shown in Fig. S2a. A crossing to anti-crossing transition containing the first EP at the critical point of $(f_{EP,exp}, td_{EP,exp}) = (0.68\,THz, 2.6\,ps)$ is clearly observable in the time-resolved eigenvalue curves. The second EP occurs at the frequency of 0.68 THz and the time delay of 8.9ps in opposite eigentransmission transition. The uncrossed eigentransmission magnitude in the regime of $\sigma < \sigma_{EP}$ at 0 ps and 10.6 ps comes from experimental errors and the frequency resolution. Similarly, numerically extracted eigentransmission magnitudes and phases with time

delay from 0 to 14.9 ps are shown in Fig. S2b. The EPs appear at the critical points of $(f_{EP,sim}, td_{EP,sim}) = (0.64\,THz, 4.9\,ps)$ and $(f_{EP,sim}, td_{EP,sim}) = (0.64\,THz, 11.5\,ps)$.

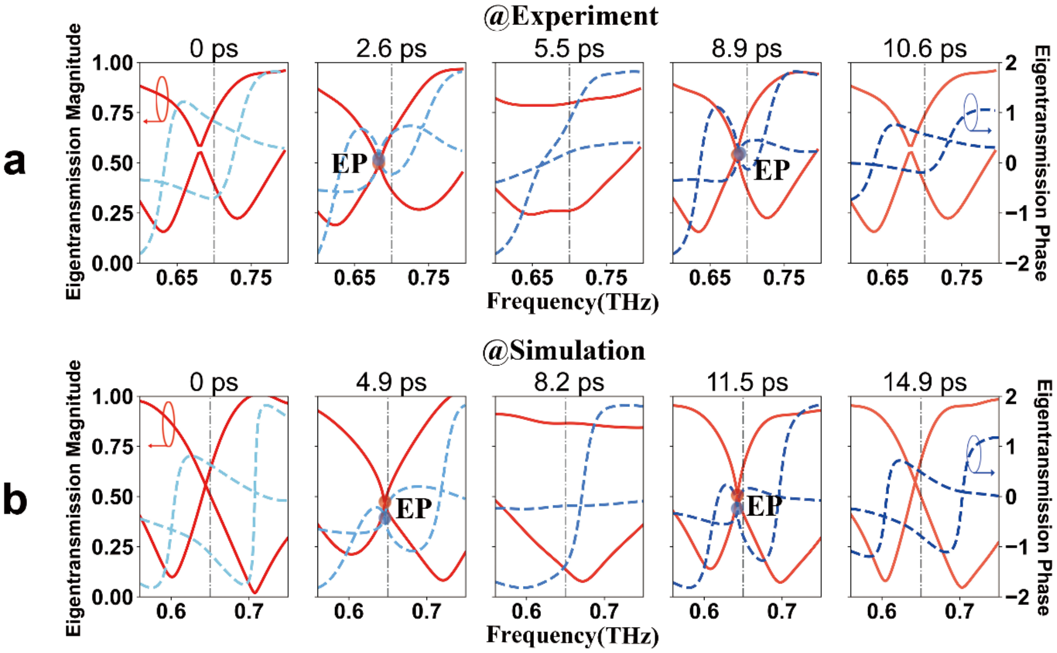

Fig. S2 *Extracted eigentransmission magnitudes and phases versus time delay from experimental and simulated eigenvalue Riemann surfaces.* (a) The evolution process of the experimental measured eigentransmission magnitude (red solid lines) and phase (blue dashed lines) with time delays of 0, 2.6, 5.5, 8.9, and 10.6 ps. The chain lines mark the frequency of 0.7 THz. EPs are experimentally found at the time delays of 2.6 and 8.9 ps. (b) The evolution process of the numerical eigentransmission magnitude (red solid lines) and phase (blue dashed lines) with time delays of 0, 4.9, 8.2, 11.5, 14.9 ps. The chain lines mark the frequency of 0.65 THz.

Temporal evolution dynamics of the chiral transmission in the Ge-hybrid metasurface implemented by transient simulation

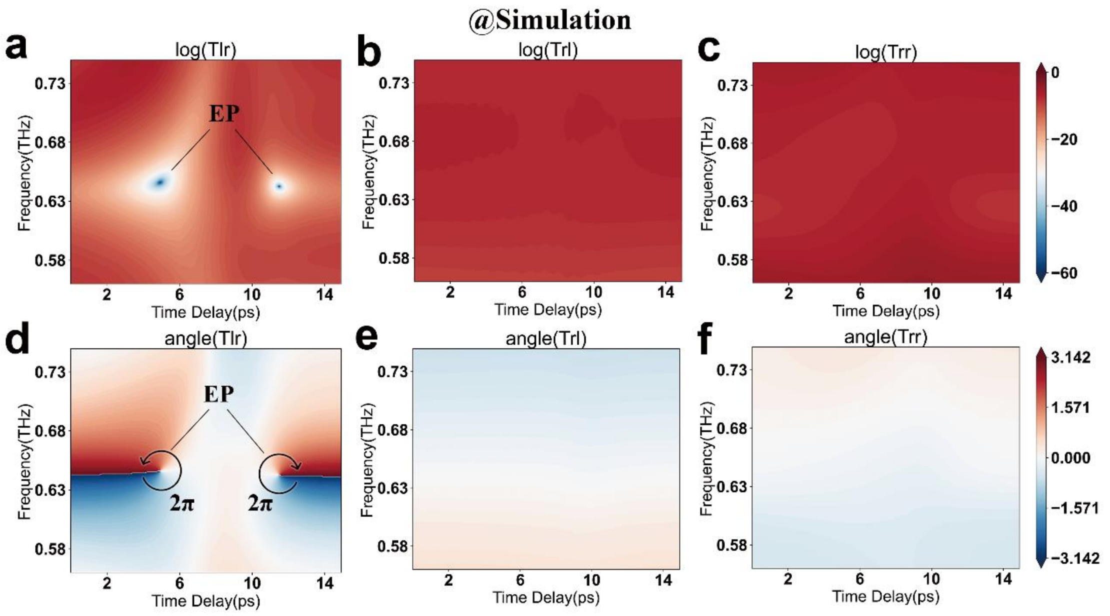

Fig. S3 *Temporal evolution dynamics of the chiral transmission in the Ge-hybrid metasurface implemented by transient simulation.* (a, b, c) The numerically simulated transmission magnitude of T_{lr} (a), T_{lr} (b), and T_{rr} (c) as a function of pump-probe time delay. (d, e, f) The numerically simulated transmission phase of T_{lr} (a), T_{lr} (b), and T_{rr} (c) as a function of pump-probe time delay. The metasurface system at EP shows asymmetric transmission, $\log(T_{lr}) \neq \log(T_{rl})$, and only the RCP component is transmitted for the input state with RCP, $\text{abs}(T_{lr}) = 0, \text{abs}(T_{rr}) \neq 0$.

Optical-pump terahertz-probe system and experimental scheme

Femtosecond plus laser in the optical path is generated by a Ti: sapphire regenerative amplifier system with 1 kHz repetition at a central wavelength of 800 nm. The terahertz generation and detection are completed by 1-mm-thick <110> ZnTe crystal. For measurement of polarization conversion when a terahertz wave passes through the Ge-hybrid non-Hermitian metasurface, one can obtain four linearly polarized components by rotating the sample and LP_2. Besides, LP_1 and LP_4 are used to ensure that the terahertz wave is polarized in the y direction for emission and detection. By rotating LP_2 so that the polarization direction follows the x and y directions respectively, the transmission components of E_x and E_y can be obtained. And rotating the sample can represent the incident in two directions. The polarization direction of LP_3 is at a 45-degree angle to the y-axis, ensuring that both E_x and E_y can be detected. The terahertz spot with a diameter of 3 mm is focused on a $5mm \times 5mm$ sample surface through a pair of parabolic mirrors. The pump light with a diameter of 5 mm is used to excite the conductivity of Ge film for modulation of the active non-Hermitian metasurface.

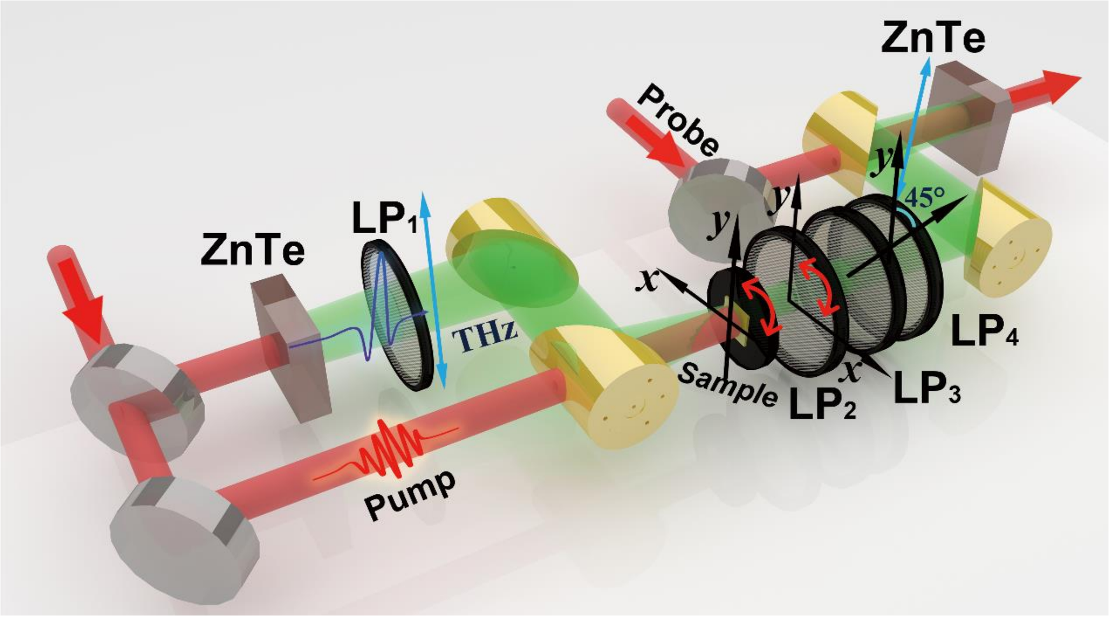

Fig. S4 A homemade optical pump terahertz-probe system for measurement of Ge-hybrid non-Hermitian metadevice. Four linearly polarized transmission signals were measured by rotating the sample and linear polarizer. LP: terahertz wire grid polarizer for linear polarization transmission.
